# Hepatic Veno-Occlusive Disease and Colorectal Cancer: Expect the Unexpected

**DOI:** 10.3390/life14070845

**Published:** 2024-07-04

**Authors:** Doina Georgescu, Daniel Florin Lighezan, Ana Lascu, Roxana Buzas, Alexandra Faur, Ioana Ionita, Ciprian Ilie Rosca, Ioana Suceava, Despina Calamar-Popovici, Mihai Ionita, Oana Elena Ancusa

**Affiliations:** 1 Department of Internal Medicine I, “V Babes” University of Medicine and Pharmacy, 300041 Timisoara, Romania; doina.georgescu@umft.ro (D.G.); dlighezan@umft.ro (D.F.L.); buzas.dana@umft.ro (R.B.); ioana.ionita@umft.ro (I.I.); rosca.ciprian@umft.ro (C.I.R.); suceava.ioana@umft.ro (I.S.); despina.calamar.popovici@umft.ro (D.C.-P.); ionita.mihai@umft.ro (M.I.); ancusa.oana@umft.ro (O.E.A.); 2 Department of Functional Sciences, “V Babes” University of Medicine and Pharmacy, 300041 Timisoara, Romania; 3 Department of Anatomy and Embriology, “V Babes” University of Medicine and Pharmacy, 300041 Timisoara, Romania; faur.alexandra@umft.ro

**Keywords:** hepatic veno-occlusive disease, portal hypertension syndrome

## Abstract

Sinusoidal obstruction syndrome/veno-occlusive disease (SOS/VOD) is a rare liver vascular condition, potentially life-threatening, with clinical signs of portal hypertension, frequently reported in relation to bone marrow transplantation and possibly in non-transplantation-related chemotherapy. We report the case of a 65-year-old female patient who insidiously developed fatigue, mild tenderness of the right upper abdominal quadrant, hepato-splenomegaly and slight weight gain consecutive to ascites development, as well as persistent elevation of transaminases and mild thrombocytopenia. To note, she had a previous history of colorectal cancer (CRC) with liver metastases and several courses of chemotherapy. Abdominal duplex and elastography measurements made the diagnosis of cirrhosis improbable. A lot of lab work-ups were performed in order to rule out several diseases and conditions. Further, transjugular access was used to perform the measurement of the hepatic venous pressure gradient and liver biopsy that confirmed SOS/VOD. In late 2023, she was diagnosed with endometrial adenocarcinoma, requiring chemotherapy again. At present, the liver condition is stationary, but the prognosis is, however, uncertain. In conclusion, we presented the atypical case of a female patient who developed portal hypertension syndrome associated with the late onset of SOS/VOD, after 5-fluorouracil and oxaliplatin chemotherapy for CRC and liver metastases, subsequently diagnosed with endometrial adenocarcinoma, which posed many diagnostic and therapeutic challenges. Given the potentially bad outcome, an early diagnosis of SOS/VOD in patients receiving drugs of risk is important not only to stratify further risk, but also to initiate an appropriate therapy in order to improve the prognosis.

## 1. Introduction

Portal hypertension syndrome, characterized by elevation in the pressure in the portal vein system, has various etiologies and pathogenies. Depending on the location of the obstacle, portal hypertension syndrome is classified into presinusoidal, sinusoidal or post-sinusoidal, being associated with a multitude of diseases and conditions [1,2]. The most frequent cause of portal hypertension and its sequels remains, however, liver cirrhosis [3,4,5,6]. 

Sinusoidal obstruction syndrome/veno-occlusive disease (SOS/VOD) is a rare liver vascular condition, potentially life-threatening, represented by a post-sinusoidal obstacle, resulting in portal hypertension, which is often reported days or weeks after chemotherapy for bone marrow transplantation (BMT). The incidence of SOS/VOD related to BMT is reported in up to 15 percent of adults, but it may vary depending on the diagnostic criteria, pediatric vs. adult patients, the study design, etc. [7]. SOS/VOD is a clinical syndrome in which a lot of risk factors may be involved. After BMT, the risk factors for SOS/VOD development are related to transplantation per se (allograft vs. autologous BMT, high doses of Busulfan) and patient and disease particularities (female gender, children vs. adults, elderly age, previous radiotherapy, metabolic syndrome, infections including viral ones, antibiotics, total parenteral nutrition, pulmonary and kidney failure, platelet refractoriness condition and poor ECOG performance status). Previous liver conditions related to chemotherapy or abdominal irradiation, active liver disease and cytolytic syndrome may also represent risk factors for SOS/VOD [8,9]. 

Apart from SOS/VOD secondary to BMT, there is growing evidence of increased risk of VOD/SOS after chemotherapy with oxaliplatin and 5-fluorouracil regimens for colorectal carcinoma (CRC). Development of SOS/VOD in relation to oxaliplatin chemotherapy for CRC is considered to be a relatively long, chronic process that may last for several months, which may lead to a late onset of SOS/VOD in CRC patients treated with oxaliplatin. These therapeutical regimens may generate toxic metabolites that will mainly hit zone 3 of the hepatic acinus, represented by hepatocytes and subsequently activated sinusoidal endothelial cells (SECs). Activated sinusoidal cells may result in hepatic sinusoidal dilation and hemorrhage. This regional cell’s swelling-up is linked to the appearance of sinusoidal barrier fragmentation. Further, the local recruitment of blood cells and the build-up of cellular debris will promote regional architectural distortions. These newly built-up products will cross the gaps between endothelial cells into the Disse spaces and aggravate the alterations in the endothelial lining. The reduced sinusoidal venous outflow will ultimately result in post-sinusoidal portal hypertension and its consequences [10]. 

In this process, liver nodules, as an expression of regenerative hyperplasia, will also occur. SOS/VOD, as a liver injury model associated with oxaliplatin chemotherapy, may clinically manifest with abnormal liver function, portal hypertension, splenomegaly and thrombocytopenia [11]. The pathology of SOS/VOD is macroscopically characterized by the so-called ‘blue liver’ aspect determined by the hepatic sinusoidal dilation. The hallmark of this liver condition is microscopically represented by the sinusoidal endothelial cell injury, together with parenchymal and venular lesions, as well as fibrotic venular occlusion [12]. 

Several pathogenic factors may contribute to SOS/VOD features. Oxaliplatin-related liver toxicity is linked in the first instance to the cytochrome P450 system, where the toxic drug is metabolized exclusively, which will trigger the severe depletion of glutathione and depolymerization of F-actin that leads to the disassembly of the actin cytoskeleton. Further release of cytokines and inflammatory cells will consecutively promote the alterations in SECs with dilation, dehiscence and ultimately obstruction of the blood flow, which, together with the activation of hepatic stellate cells, will reshape the whole acinar area. In the end, abundant collagen bundles will be seen in the perisinusoidal spaces and a fibrotic venular occlusion will develop [13]. 

A background of chemotherapy related to BMT or CRC, clinical signs of portal hypertension, characterized by hepatomegaly, jaundice, thrombocytopenia and ascites with weight gain should suggest SOS/VOD [14]. Diagnosis of SOS/VOD is currently based on the Seattle and Baltimore criteria, but none of them are suitable either for the early diagnosis setting or for the late-onset variant of SOS/VOD [15]. The revised criteria published in 2020 added useful clarifications for SOS/VOD diagnosis [16]. 

Imaging studies such as 2D ultrasonography and abdominal duplex examination are widely used in the SOS/VOD approach. In the 2D mode of liver ultrasonography, the following features may be observed: hepatomegaly/splenomegaly, gallbladder wall thickness, ascites in various amount, vascular modifications with narrowing of hepatic veins and dilatation of main portal vein with collateral circulation. Doppler-mode ultrasound gives information about portal vein system hemodynamics. Spectral demodulation, alterations in velocities and flow direction, increase in the resistivity index of the hepatic artery and modifications of the hepatic veins’ flow with monophasic spectral aspects are often observed. In a contrast-enhanced ultrasound, a hypo-enhancement of the liver parenchyma may be noted. The elastography may confirm a low liver stiffness and helps to exclude cirrhosis. All these liver features described by the abdominal ultrasound are considered to be highly reliable for SOS/VOD diagnosis. CT/MRI displays no other specific imaging signs in SOS/VOD [17]. The outcome of SOS/VOD can range from latent, oligosymptomatic liver disease, to various degrees of liver damage with the development of portal hypertension syndrome. Some cases may display a severe evolution with acute multiple organ failure and poor prognosis [18]. Related to therapy options, some studies have reported that a high dose of methylprednisolone alone or associated with Defibrotide (DF) has mitigated the clinical and biological expression of SOS/VOD. However, at present, DF is the only agent recommended for the treatment of severe SOS/VOD [19]. 

## 2. Case Report

**Figure 1:**
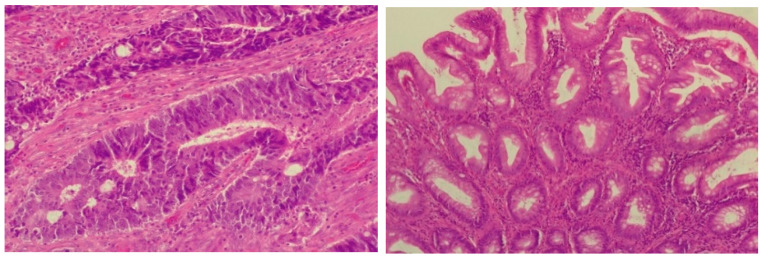
Adenocarcinoma showing moderately differentiated glands filled with necrotic debris. HE stain, 400× magnification (**left**); hyperplastic polyp with “sawtooth” appearance, mild nuclear enlargement and no horizontal crypts, HE stain, 200× magnification (**right**).

A 65-year-old female was admitted to the outpatient department in late 2023, complaining about the insidious onset of fatigue, slight abdominal pain and weight gain. The patient’s medical history highlighted a rectal adenocarcinoma (T2N1M0) diagnosed in 2014, treated by surgery, chemotherapy (4× FUFOL) and radiotherapy. The histology of the adenocarcinoma is presented below, in Figure 1.

Two years later, she was diagnosed with segment VII liver metastasis and underwent segmentectomy and adjuvant chemotherapy (Cape OX). A few years later, the patient experienced one episode of upper digestive bleeding by variceal effraction treated by endoscopic ligations (2020). The upper digestive endoscopy performed on this occasion detected esophageal varices grade I/II and portal hypertension gastropathy. At that time, her liver tests showed only a moderate cytolytic syndrome. Therefore, she received a treatment with nonselective betablockers and spironolactone. Given the pandemic situation, further appointments with a gastroenterologist and other work-ups or procedures were, however, postponed.

**Figure 2:**
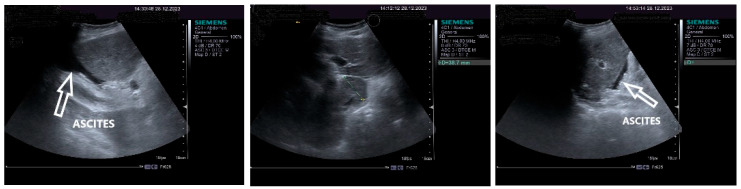
The 2D ultrasound aspect of the liver: perihepatic ascites (**right**,**left**), caudate lobe at the upper limit (**middle**).

On physical examination, a mild tenderness of the right upper abdominal quadrant, a firm hepatomegaly, a palpable inferior pole of the spleen and a slight increase in abdomen volume were found. No other liver stigmata were noticed. The abdominal duplex ultrasound examination, performed with Siemens high-resolution equipment, showed hepatomegaly with a discrete irregularity of the liver surface, heterogeneity of the liver texture, the caudate lobe at the upper limit, filiform hepatic veins, massive thickening of the gallbladder wall, ascites in small quantity and mild splenomegaly with turbulence of the venous flow in the hilum. The question about liver cirrhosis was asked. Various aspects of the 2D ultrasound examination of the liver are depicted in Figure 2.

**Figure 3:**
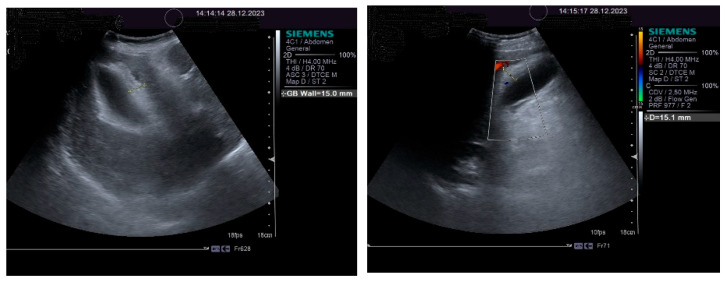
Thickening of gallbladder wall: 2D aspect (**left**); color Doppler aspect (**right**).

The impressive thickening of the gallbladder wall is illustrated in Figure 3.

**Figure 4:**
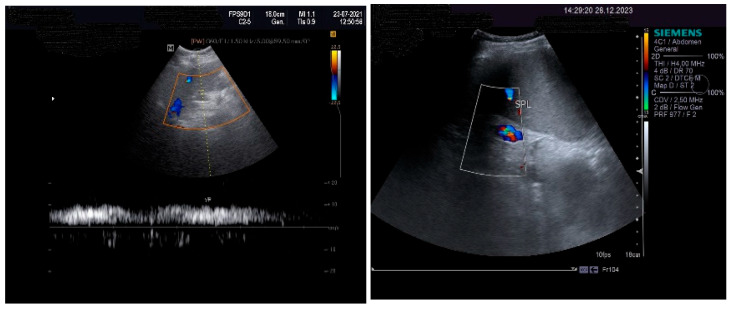
Doppler examination. Liver: pulsed Doppler of the portal vein (**left**). Spleen: color Doppler of splenic vein (**right**).

The pulsed Doppler ultrasound revealed a patency of the main portal vein as well as of the left and right portal branches and decreased, demodulated hepatopetal portal venous flow, with mildly decreased respiratory variability in the amplitude of the portal vein flow. The Dopler aspects of the liver and spleen are illustrated in Figure 4.

**Figure 5:**
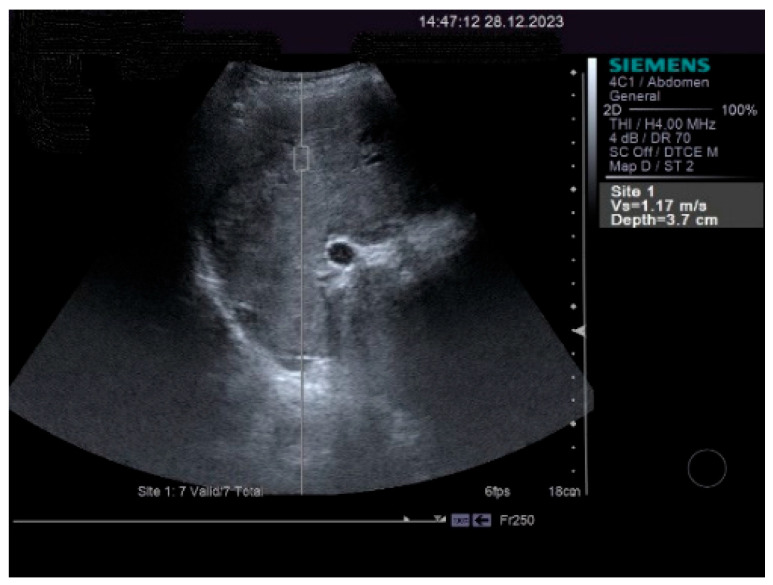
p SWE elastography of the right liver lobe.

The measurements of the resistivity index of the hepatic artery at the liver hilum revealed increased values (HARI = 0.79). However, the liver elastography demonstrated a low stiffness. The measurements were made using the acoustic radiation force impulse (ARF)I method, by point shear wave (p SWE) elastography, and the obtained median value was 1.24 m/s, IQR = 0.12, equivalent to an F2 Metavir fibrosis score. Therefore, the diagnosis of liver cirrhosis was highly improbable. The elastography of the right liver lobe performed by the ARFI method, pSWE, is presented in Figure 5.

Her baseline lab work-ups displayed mild elevation of the transaminases with ALAT = 58 IU/dL, ASAT = 62 IU/dL, de Rittis ratio = 1.06, moderate thrombocytopenia = 115.000/mm^3^, APRI = 2.1 and FIB4 = 4.6. The other routine tests were within normal range: hemoglobin = 12.3 g/dL, leukocytes = 6700/mm^3^, gamma-GT = 22 U/L, alkaline phosphatase = 42 U/L, total bilirubin = 0.46 mg/dL, ELFO: total proteins = 6.3 g/dL, albumin = 62%, alpha1 globulins = 4.5%, alpha2 globulins = 10.5%, beta 1 globulins = 6.5%, beta 2 globulins = 5.5%, gamma globulins = 14.5%, INR = 1.1, C-reactive protein = 4.8 mg/L, fasting plasma glucose = 82 mg/dL, Hb A_1_C = 5.4%, creatinine = 0.68 mg/dL and uric acid = 5.4 mg/dL. HBs antigen, antibodies anti-Delta and anti-HCV were absent, plasma iron = 71.3 µg/dL and copper = 72.4 µg/dL, alpha 1 antitrypsin = 1.3 g/L^3^, alpha-fetoprotein = 3.33 ng/mL, as well as total cholesterol = 185 mg/dL, low-density lipoprotein = 88 mg/dL and high-density lipoprotein = 68 mg/dL, triglycerides = 115 mg/dL. In order to rule out several diseases and conditions, further tests were run. The antinuclear antibodies, anti-smooth muscle antibodies, antimitochondrial antibodies, the QuantiFERON (TB Gold Plus) test and the angiotensin-converting enzyme were within normal range.

**Figure 6:**
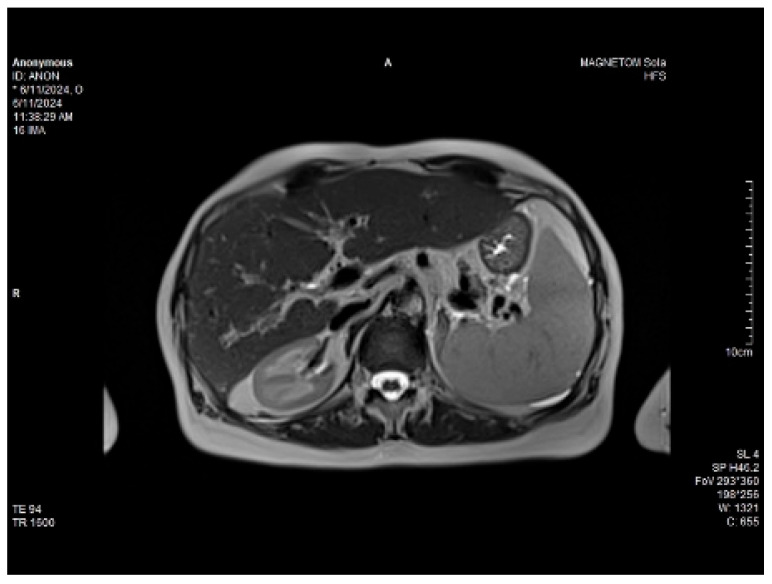
MRI featuring perihepatic and perisplenic ascites.

**Figure 7:**
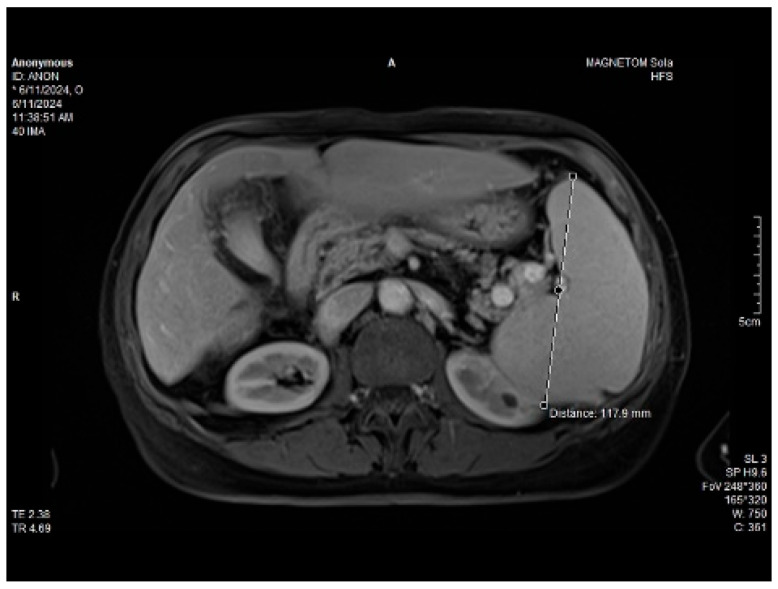
MRI: splenomegaly.

**Figure 8:**
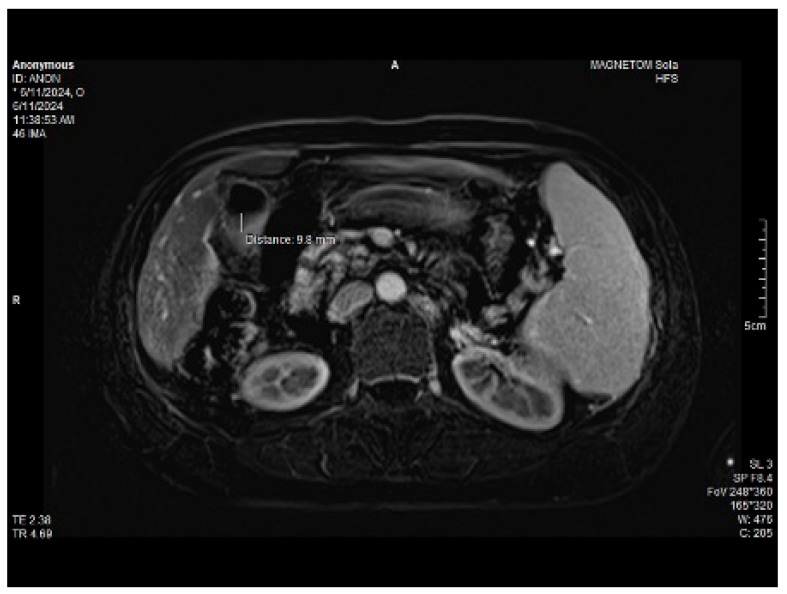
Massive thickening of the gallbladder wall, MRI features.

**Figure 9:**
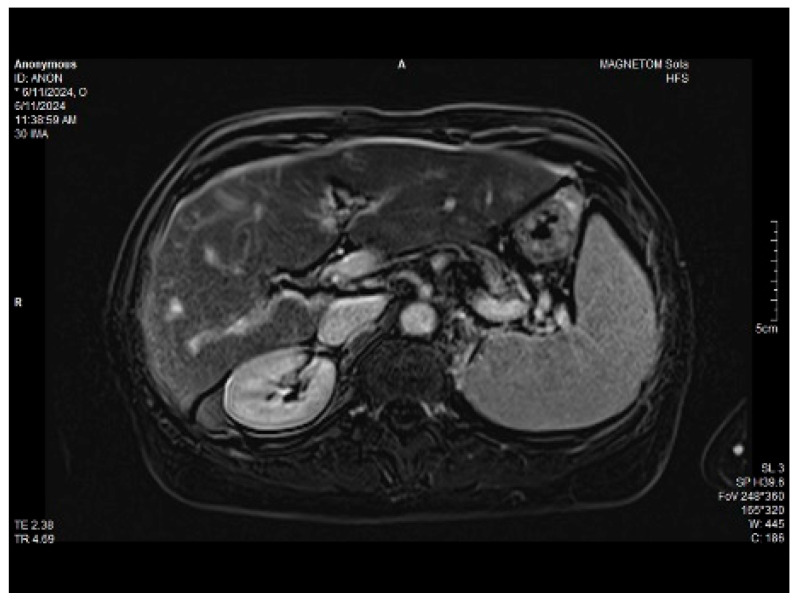
Small veno-portal shunts visible at the periphery of both hepatic lobes: MRI aspects.

Abdominal MRI study confirmed the hepato-splenomegaly, ascites and the patency of the portal vein system. In addition, small veno-portal shunts were highlighted at the periphery of both hepatic lobes. Various MRI features are depicted in Figure 6, Figure 7, Figure 8 and Figure 9.

**Figure 10:**
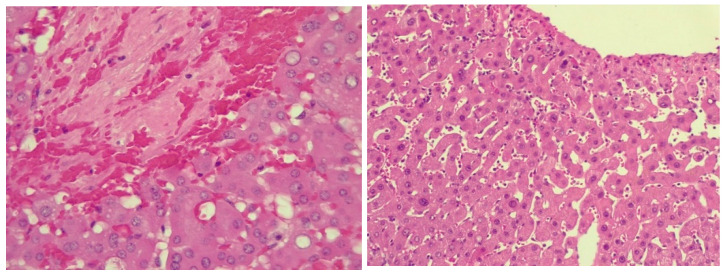
Liver swing areas with congestion, hemorrhagic necrosis, isolated atrophy of hepatocytes and occlusion of terminal hepatic venule, HE stain, 400× magnification (**left**); liver showing markedly dilated sinusoids and central vein, HE stain, 200× magnification (**right**).

Because the MRI imaging study did not bring relevant data for the diagnosis and still had the suspicion of the late onset of SOS/VOD after chemotherapy for CRC, transjugular access was used to measure the hepatic venous pressure gradient (HVPG) and to perform a safe liver biopsy. The puncture of the right jugular vein was performed under ultrasound guidance. The HVPG was measured at the level of all hepatic veins, with the average result being 10.6 mmHg. Liver biopsy was performed in a single passage, by the transjugular approach through the right hepatic vein wall, with a Colapinto needle. The liver specimen was not fragmented, and had a total length of 47 mm with a thickness of 1 mm. In order to perform the histopathological analysis, the liver fragment was fixed in 10% formalin for 24 h, embedded in paraffin and sectioned into 3 μm thick and 4 μm thick multiple sections using a manual rotary Leica microtome. The samples were stained with hematoxylin and eosin (HE), trichrome Masson, Perls stain for iron and Rhodamine stain for copper. The obtained slides were studied using a Leica DM750, microscope (Leica Microsystems Schweiz AG, Heerbrugg, Switzerland), with a digital camera (200× and 400× magnification). The liver specimen contained about 30 portal spaces. Most portal spaces displayed reduced inflammatory infiltrate made up of lymphocytes and neutrophils, with focal interface activity. Several portal spaces showed venopenia and 2–3 periportal shunt vessels were detected. In two portal spaces, epithelioid granulomas were sketched. Hepatocytes had reduced mixed steatosis and rare glycogenated nuclei. Focal sinusoidal dilatations were present. Trichrome Masson staining revealed reduced focal perisinusoidal fibrosis and minimal portal fibrosis. Staining for iron and copper were negative. Liver parenchyma presented with focal vascular disorders, hepatocyte centrilobular apoptosis, necrosis and viable periportal hepatocytes consistent with SOS/VOD diagnosis. Histologic aspects of the liver samples stained by HE are represented in Figure 10. 

The certainty diagnosis was SOS/VOD of mild severity, with late-onset and atypical clinical presentation. In the context of a mild hepatic SOS/VOD, DF therapy was not taken into discussion. The patient pursued her previous therapeutical schedule with nonselective betablockers and spironolactone. Due to overlapping of recent gynecological complaints and coincidental elevation of some tumoral markers, such as CEA = 134 ng/mL and CA19-9 = 646 U/mL^3^, an abdominal and pelvic MRI were ordered and performed in late 2023. The imaging study revealed a uterine mass and nearby enlarged lymph nodes. Consecutively, a uterine and lymph node biopsy procedure was performed. The pathology analysis confirmed endometrial adenocarcinoma with regional lymph nodes invasion, T2N1M0, which prompted another oncological course of treatment. At present, the patient pursues her oncological treatment plan for the gynecological malignancy and her liver status is under close supervision, being treated by nonselective betablockers, spironolactone and liver supportive therapy. Monthly liver work-ups are carefully followed up. For now, her liver condition is stationary, but due to recent developments related to second malignancy, the prognosis is, however, uncertain.

## 3. Discussions 

The concept of SOS/VOD evolved over the years from the first described case of „obliterative endophlebitis in the terminal hepatic veins” at the beginning of the 20th century, to nowadays, when this liver condition is well characterized and defined as a distinguished lesion, apart from Budd–Chiari and Banti syndromes [20]. The precise frequency of SOS/VOD is difficult to estimate given the multitude of involved variables, such as type of BMT, therapeutical regimen and underlying liver conditions. The overall incidence of SOS/VOD has a wide range from 5 to 60% in children and adults as well. However, retrospective studies reported many cases of underdiagnosed SOS/VOD, especially in cases with the late onset of the liver condition [21]. From the clinical standpoint, there are some differences regarding the specific symptoms and signs of the early onset vs. the late onset of SOS/VOD. In contrast to the early onset of SOS/VOD, where jaundice is almost ubiquitously present, the diagnostic criteria are clearly stated and access to Defibrotide therapy is rapidly granted, the late onset of the same condition does not share all the clinical aspects. In this particular situation, related to the subacute and chronic forms of SOS/VOD, the onset of the symptoms may take weeks, months or even years after chemotherapy [22,23]. As we have emphasized in the current case report, the late onset of SOS/VOD may lack hyperbilirubinemia and jaundice, the patient being anicteric. Other symptoms and signs suggesting a liver disease may initially be discrete. In this context, an early diagnosis is often difficult and therefore a delay in the specific treatment is frequently reported with prognostic consequences.

Several chemotherapeutical agents against cancer, such as alkylating agents (busulfan, cyclophosphamide dacarbazine) or platinum coordination complexes (carboplatin, cisplatin and oxaliplatin) may cause SOS/VOD. Based on clinical trial data, various chemotherapy regimens used for CRC treatment may exhibit a high risk of SOS/VOD. In this context, prophylaxis and the limitation of SOS/VOD risk was stated on an expert consensus panel that recommended ≤2 inotuzumab or ozogamicin treatment cycles. Since oxaliplatin (OX), acting as a DNA synthesis inhibitor, was systematically used in the treatment against CRC, many side effects represented by gastrointestinal symptoms were recorded. Among these side effects, SOS/VOD turned out to be an important latent issue and apparently less appreciated [24]. In rare cases, SOS/VOD may develop after high-dose therapy with thiopurine agents, represented by azathioprine, mercaptopurine and thioguanine. Exposure to pyrrolizidine alkaloids found in some weeds or various plants may sometimes be associated with liver SOS/VOD [24]. Several pathogenic pathways may be involved in liver damage. Direct toxicity related to various chemotherapeutical agents may result in the depletion of the antioxidant compound from hepatocytes such as glutathione and increase sensitivity to acinar zone 3 injury. The endothelial injury will trigger the release of proinflammatory cytokines TNF-α, IL1 and IL2, increase production of vascular endothelial growth factor and activation of clotting factors with the early deposition of matrix metalloproteinases-2 and late deposition of matrix metalloproteinases-9. The decrease in the hepatic venous flow due to architectural rearrangement will favor the local decline in the nitric oxide levels, resulting in vasoconstriction that aggravates portal hypertension syndrome [25]. The patient we have presented received several courses of chemotherapeutical agents, FUFOL and CapeOX, due to CRC and further liver metastases, which placed her in the category of high risk of SOS/VOD development. Given that several studies frequently reported oxaliplatin as a key agent causing sinusoidal damage and triggering the onset of SOS/VOD, with increased morbidity before or after liver surgery, it became more and more obvious that a prophylactic approach might be needed. 

Apart from chemotherapy, a role in SOS/VOD development is also represented by the genetic predisposition, an autosomal recessive condition with immunodeficiency, as well as by the secondary susceptibility of the liver in patients with a history of previous liver conditions such as viral hepatitis, toxic exposure or radiation treatment. Whether previous abdominal radiotherapy performed years ago to the presented patient, as a part of the oncological treatment algorithm against CRC, has increased the liver’s susceptibility to SOS/VOD, it was a scenario that we could have taken into account. However, the SOS/VOD onset described after radiotherapy is mostly associated with hepatic radiation. The severity of the liver condition depends on the amount of the radiation’s total dose, the functional liver capabilities and the patient’s medical history [26].

Given the variability in the clinical picture, ranging from a mild liver condition to life-threatening situations, the optimization of the SOS/VOD diagnosis is an important task to fulfill. Therefore, severity biomarkers have been proposed as an asset for clinicians in order to improve the management of SOS/VOD. The endothelial activation and stress index (EASIX) is a biomarker panel that uses lactate dehydrogenase, creatinine and thrombocytes that might be promising in identifying the populations at high risk of developing severe SOS/VOD [27].

An important role in the imaging diagnostic approach of SOS/VOD is played by ultrasonography. According to some studies, it seems that ascites and gallbladder wall edema represent independent predictors in SOS/VOD diagnosis. Some authors reported that the thickness of the gallbladder wall correlated well with the HVPG. This particular aspect of the markedly thickened GB wall was also observed in the presented patient, associated with ascites and increase in the HVPG. Pulsed Doppler ultrasound is also useful for suggesting SOS/VOD, based on the study of the venous portal flow, which can be decreased or reversed. A significant increase in the HARI may also be associated with liver injury. Hepatic and portal flow anomalies revealed by duplex examination seem to correlate with the HVPG. However, as we have also noted, the reversed portal venous flow is not always recorded and, therefore, one cannot exclude SOS/VOD based on the absence of the hepatofugal portal flow sign [28]. The patient that we have presented in this case report displayed no reversed portal venous flow, but increased values of the HARI. 

The severity assessment of SOS/VOD has critical importance for clinicians. The currently implemented grading system for the adult population is represented by categories related to severity, as follows: grade 1 = mild, grade 2 = moderate, grade 3 = severe, grade 4 = very severe [29,30]. The patient we have presented displayed a tableau consistent with a mild form of SOS/VOD, while ASAT and ALAT were < 2×, persistent refractory thrombocytopenia lasted < 3 days, bilirubin was within normal range, ascites had a minimal amount and coagulation was normal, as well as the glomerular filtration rate, pulmonary function and central nervous system. Even if at present the patient exhibited a mild form of SOS/VOD, it is difficult to draw her future evolution. Therefore, she should be closely monitored. 

Although the outcome of SOS/VOD is generally good, with many patients succeeding in recovering, the prognosis, however, may be unpredictable. There is a wide debate related to the possible reversibility of sinusoidal injury in SOS/VOD. Some studies conducted in human and animal models demonstrated the mitigation of liver damage after chemotherapy cessation, while others reported the persistence or progression of pathological features, even after limitation to toxic exposure [31,32]. 

Therapy with DF is recommended mostly in the treatment of moderate/severe forms of SOS/VOD and less in its prevention. Other therapies such as anticoagulation and thrombolytic therapies are frequently associated with a significant bleeding risk. Some studies reported a good response to high doses of methylprednisolone [33]. Given that therapy for SOS/VOD is not successful all the time, especially in severe cases, a prophylactic approach may be useful. Ursodeoxycholic acid (UDCA) was evaluated in many studies for its preventive capabilities. UDCA can modulate cytokines expression and therefore interfere with inflammation. It may be useful prior to BMT or at the initialization of chemotherapy for metastatic CRC. It seems that the administration of UDCA for at least 3 months after BMT may significantly reduce severe liver damage and other potentially life-threatening complications. Low doses of heparin or LMWH may also help to prevent SOS/VOD [34]. DF was used in children and adult patients not only as a therapeutical agent against SOS.VOD, but also as preventive therapy, with promising results. Some studies reported that DF was effective and well tolerated. A low rate of side effects and reduced number of dropped-out cases were reported after the prevention of SOS/VOD with DF [35]. Due to the mild clinical form of SOS/VOD, the presented patient was not a suitable candidate for DF treatment, so she received nonselective betablockers, spironolactone and liver supportive therapy, being carefully clinically and biologically followed up. Even if her liver disease is currently mild and so far, the outcome was satisfactory, the prognosis remains, however, uncertain. To note, the gynecological malignancy is an additional burden for a fragile liver condition. She had to go back to chemotherapy, possible radiotherapy and another surgical procedure. All these oncological steps may unfavorably influence her liver status and should be carefully managed.

## 4. Conclusions

In conclusion, we presented the atypical case of a female patient who developed portal hypertension syndrome associated with the late onset of SOS/VOD, after 5-fluorouracil and oxaliplatin chemotherapy for CRC and liver metastases, subsequently diagnosed with endometrial adenocarcinoma, which posed many diagnostic and therapeutic challenges. Given the potentially bad outcome, an early diagnosis of SOS/VOD in patients receiving drugs of risk is important not only to stratify further risk, but also to initiate an appropriate therapy in order to improve the prognosis.

## Data Availability

Data will be provided upon written request.

## References

[1] Bosch J., Iwakiri Y. (2018). The portal hypertension syndrome: Etiology, classification, relevance, and animal models. Hepatol. Int..

[2] Brown S.A., Trinh V.Q., Healan S., Bichell D., Frischhertz B., Scanga A. (2021). An Anomalous Cause of Portal Hypertension. ACG Case Rep. J..

[3] La Mura V., Nicolini A., Tosetti G., Primignani M. (2015). Cirrhosis and portal hypertension: The importance of risk stratification, the role of hepatic venous pressure gradient measurement. World J. Hepatol..

[4] Berzigotti A. (2017). Advances and challenges in cirrhosis and portal hypertension. BMC Med..

[5] Georgescu D., Iurciuc M., Ionita I., Georgescu L.A., Muntean M., Lascu A., Ionita M., Lighezan D. (2019). Portal vein thrombosis and gut microbiota: Understanding the burden. Rev. Chim..

[6] Iwakiri Y., Trebicka J. (2021). Portal hypertension in cirrhosis: Pathophysiological mechanisms and therapy. JHEP Rep..

[7] Mohty M., Malard F., Alaskar A.S., Aljurf M., Arat M., Bader P., Baron F., Bazarbachi A., Blaise D., Brissot E. (2023). Diagnosis and severity criteria for sinusoidal obstruction syndrome/veno-occlusive disease in adult patients: A refined classification from the European society for blood and marrow transplantation (EBMT). Bone Marrow Transplant..

[8] Dalle J.H., Giralt S.A. (2016). Hepatic Veno-Occlusive Disease after Hematopoietic Stem Cell Transplantation: Risk Factors and Stratification, Prophylaxis, and Treatment. Biol. Blood Marrow Transplant..

[9] Yoon J.H., Choi C.W., Won J.H. (2021). Hepatic sinusoidal obstruction syndrome/veno-occlusive disease after hematopoietic cell transplantation: Historical and current considerations in Korea. Korean J. Int. Med..

[10] Mohty M., Malard F., Abecassis M., Aerts E., Alaskar A.S., Aljurf M., Arat M., Bader P., Baron F., Bazarbachi A. (2015). Sinusoidal obstruction syndrome/veno-occlusive disease: Current situation and perspectives-a position statement from the European society for blood and marrow transplantation (EBMT). Bone Marrow Transplant..

[11] Zhu C., Ren X., Liu D., Zhang C. (2021). Oxaliplatin-induced hepatic sinusoidal obstruction syndrome. Toxicology.

[12] Calistri L., Rastrelli V., Nardi C., Maraghelli D., Vidali S., Pietragalla M., Colagrande S. (2021). Imaging of the chemotherapy-induced hepatic damage: Yellow liver, blue liver, and pseudocirrhosis. World J. Gastroenterol..

[13] van Mierlo K.M.C., Zhao J., Kleijnen J., Rensen S.S., Schaap F.G., Dejong C.H.C., Damink S.W.M.O. (2016). The influence of chemotherapy-associated sinusoidal dilatation on short-term outcome after partial hepatectomy for colorectal liver metastases: A systematic review with meta-analysis. Surg. Oncol..

[14] Bonifazi F., Barbato F., Ravaioli F., Sessa M., Defrancesco I., Arpinati M., Cavo M., Colecchia A. (2020). Diagnosis and Treatment of VOD/SOS After Allogeneic Hematopoietic Stem Cell Transplantation. Front. Immunol..

[15] Carreras E. (2015). How I manage sinusoidal obstruction syndrome after haematopoietic cell transplantation. Br. J. Haematol..

[16] Mohty M., Malard F., Abecasis M., Aerts E., Alaskar A.S., Aljurf M., Arat M., Bader P., Baron F., Basak G. (2020). Prophylactic, preemptive, and curative treatment for sinusoidal obstruction syndrome/veno-occlusive disease in adult patients: A position statement from an international expert group. Bone Marrow Transplant..

[17] Bohte A.E., Dierselhuis M.P., van Noesel M.M., Lequin M.H. (2022). Imaging features of hepatic sinusoidal obstruction syndrome or veno-occlusive disease in children. Pediatr. Radiol..

[18] Lewis C., Kim H.T., Roeker L.E., Cutler C., Koreth J., Nikiforow S., Armand P., Gootpu M., Romee R., Glotzbecker B. (2020). Incidence, Predictors, and Outcomes of Veno-Occlusive Disease/Sinusoidal Obstruction Syndrome after Reduced-Intensity Allogeneic Hematopoietic Cell Transplantation. Biol. Blood Marrow Transplant..

[19] Tewari P., Wallis W., Kebriaei P. (2017). Manifestations and Management of Veno-occlusive Disease/Sinusoidal Obstruction Syndrome in the Era of Contemporary Therapies. Clin. Adv. Hematol. Oncol..

[20] Fan C.Q., Crawford J.M. (2014). Sinusoidal obstruction syndrome (hepatic veno-occlusive disease). J. Clin. Exp. Hepatol..

[21] Bazarbachi A.H., Al Hamed R., Labopin M., Halaburda K., Labussiere H., Bernasconi P., Schroyens W., Gandemer V., Schaap N.P., Loschi M. (2021). Underdiagnosed veno-occlusive disease/sinusoidal obstruction syndrome (VOD/SOS) as a major cause of multi-organ failure in acute leukemia transplant patients: An analysis from the EBMT Acute Leukemia Working Party. Bone Marrow Transplant..

[22] Castellino A., Guidi S., Dellacasa C.M., Gozzini A., Donnini I., Nozzoli C., Manetta S., Aydin S., Giaccone L., Festuccia M. (2018). Late-Onset Hepatic Veno-Occlusive Disease after Allografting: Report of Two Cases with Atypical Clinical Features Successfully Treated with Defibrotide. Mediterr. J. Hematol. Infect. Dis..

[23] Mehra V., Tetlow S., Choy A., de Lavallade H., Kulasekararaj A., Krishnamurthy P., Avenoso D., Marsh J., Potter V., Mufti G. (2021). Early and late-onset veno-occlusive disease/sinusoidal syndrome post allogeneic stem cell transplantation—A real-world UK experience. Am. J. Transplant..

[24] Jafari A., Matthaei H., Wehner S., Tonguc T., Kalff J.C., Manekeller S. (2018). Bevacizumab exacerbates sinusoidal obstruction syndrome (SOS) in the animal model and increases MMP 9 production. Oncotarget.

[25] Wang Y., Qiao D., Li Y., Xu F. (2018). Risk factors for hepatic veno-occlusive disease caused by *Gynura segetum*: A retrospective study. BMC Gastroenterol..

[26] Kim J., Jung Y. (2017). Radiation-induced liver disease: Current understanding and future perspectives. Exp. Mol. Med..

[27] Ansari M., Petrykey K., Rezgui M.A., Del Vecchio V., Cortyl J., Ralph R.O., Nava T., Beaulieu P., St-Onge P., Mlakar S.J. (2020). Genetic Susceptibility to Hepatic Sinusoidal Obstruction Syndrome in Pediatric Patients Undergoing Hematopoietic Stem Cell Transplantation. Biol. Blood Marrow Transplant..

[28] Jiang S., Penack O., Terzer T., Schult D., Majer-Lauterbach J., Radujkovic A., Blau I.W., Bullinger L., Muller-Tidow C., Dreger P. (2021). Predicting sinusoidal obstruction syndrome after allogeneic stem cell transplantation with the EASIX biomarker panel. Haematologica.

[29] Skeens M.A., McArthur J., Cheifetz I.M., Duncan C., Randolph A.G., Stanek J., Lehman L., Bajwa R. (2016). High variability in the reported management of hepatic veno-occlusive disease in children after hematopoietic stem cell transplantation. Biol. Blood Marrow Transplant..

[30] Chao N. (2014). How I treat sinusoidal obstruction syndrome. Blood.

[31] Corbacioglu S., Carreras E., Ansari M., Balduzzi A., Cesaro S., Dalle J.H., Dignan F., Gibson B., Guengoer T., Gruhn B. (2018). Diagnosis and severity criteria for sinusoidal obstruction syndrome/veno-occlusive disease in pediatric patients: A new classification from the European society for blood and marrow transplantation. Bone Marrow Transplant..

[32] Lai X., Liu L., Zhang Z., Shi L., Yang G., Wu M., Huang R., Liu R., Lai Y., Li Q. (2021). Hepatic veno-occlusive disease/sinusoidal obstruction syndrome after hematopoietic stem cell transplantation for thalassemia major: Incidence, management, and outcome. Bone Marrow Transplant..

[33] Gressens S.B., Cazals-Hatem D., Lloyd V., Plessier A., Payancé A., Lebrec D., Durand F., Socie G., Valla D., Paradis V. (2022). Hepatic venous pressure gradient in sinusoidal obstruction syndrome: Diagnostic value and link with histological lesions. JHEP Rep..

[34] Fulgenzi A., Ferrero M.E. (2016). Defibrotide in the treatment of hepatic veno-occlusive disease. Hepat. Med..

[35] Strouse C., Zhang Y., Zhang M.J., DiGilio A., Pasquini M., Horowitz M.M., Lee S., Ho V., Ramanathan M., Chinratanalab W. (2018). Risk Score for the development of veno-occlusive disease after allogeneic hematopoietic cell transplant. Biol. Blood Marrow Transplant..

